# Degree of thrombocytopenia and severity of retinopathy of prematurity

**DOI:** 10.1038/s41372-026-02602-3

**Published:** 2026-03-05

**Authors:** Mohamed Mansour, Vanishree Nandakumar, Hany Aly, Mohamed A. Mohamed

**Affiliations:** 1 Cleveland Clinic Children’s, Cleveland, OH USA; 2 MetroHealth System, Cleveland, OH USA

**Keywords:** Risk factors, Predictive markers

## Abstract

**Background:**

Growing evidence links thrombocytopenia to retinopathy of prematurity (ROP) in preterm infants. However, it remains unclear whether the degree of thrombocytopenia affects the severity of ROP.

**Methods:**

VLBW (<1500 g) infants were divided into two groups. Group (A) with and Group (B) without thrombocytopenia. Chi square test, logistic regression, and correlation coefficient analysis models were used for statistical analysis. Statistical significance was set at a *p* value < 0.05.

**Results:**

Of the 305 VLBW infants, 47% developed ROP in group A compared to 16% in group B. Severe ROP occurred in 20% of infants in group A, whereas none in group B developed severe ROP. There was a significant inverse relationship between platelets count as a continuous variable and the grade of ROP (Spearman correlation coefficient: −0.36, *p* < 0.001).

**Conclusion:**

Thrombocytopenia is an independent risk factor for ROP in VLBW infants, with lower platelet counts associated with increased ROP severity.

## Introduction

Retinopathy of prematurity (ROP) is a major cause of visual impairment and potential blindness in preterm infants. This vasoproliferative disorder impacts the developing retinal vasculature and is influenced by both systemic and ocular factors associated with premature birth. The administration of supplemental oxygen, though essential in the management of preterm infants, has been identified as an important factor in the development of ROP. Fluctuations in oxygen levels are closely linked to the disorder of abnormal retinal vascularization [1]. The impact of systemic factors, notably hematological anomalies like thrombocytopenia remains an area of ongoing research.

Thrombocytopenia, commonly seen in neonatal intensive care settings, significantly affects preterm infants, with its prevalence inversely related to both gestational age and birth weight [2]. The connection between thrombocytopenia and ROP development is believed to stem from the multifaceted roles of platelets in angiogenesis and vascular regulation. Platelets are crucial for storing, transporting, and modulating the activity of angiogenic factors, such as vascular endothelial growth factor (VEGF) and insulin-like growth factor 1 (IGF-1). Both factors are important in retinal vascular development and the pathophysiology of ROP [3]. The role of platelet transfusion in developing ROP is not well studied. However, it is speculated that platelet transfusions may be associated with release of disproportionate amounts of the VEGF, IGF-1 or similar mediators that would be essential in the pathogenesis of ROP [4]. Recent studies suggested that thrombocytopenia, and consequently platelets transfusion, during the neonatal period could affect the onset and advancement of ROP through mechanisms related to altered angiogenic signaling and vascular development [5, 6]. Choręziak et al. study demonstrated a correlation between thrombocytopenia and the potential occurrence of ROP, underscoring the association of thrombocytopenic episodes in the diagnosis, development and progression of ROP [5]. This observation is supported by Jensen et al., who found a significant association between thrombocytopenia and severe ROP within specific postmenstrual age intervals, further emphasizing the role of systemic factors in the disease’s progression [6]. Additionally, recent findings from a Bayesian model-averaged meta-analysis reinforced the significance of platelet counts in the risk assessment of severe ROP, highlighting the importance of evaluating platelet levels as a potentially modifiable risk factor [7].

This study aims to build on these insights by evaluating the association of thrombocytopenia with the development of ROP and identify the association of the variability of platelets count with the degree of ROP in a cohort of VLBW infants.

## Methods

### Study design and setting

This retrospective study was conducted at one hospital system with two level III and one level IV neonatal intensive care units (NICUs). We included all VLBW infants born between January 2019 to December 2021. The study was approved by the Institutional Review Board (IRB) as part of retrospective data collection protocol of VLBW infants admitted at these NICUs. Informed consent was waived due to the retrospective nature of the study.

### Participants

Inclusion criteria were VLBW infants with a birth weight of less than 1500 g. We excluded infants with congenital anomalies affecting survival, those who did not receive an eye examination for ROP, and infants transferred from other institutions without complete medical records. Infants were divided into two groups based on their platelet counts: Group (A) included infants who developed thrombocytopenia (platelet count <150,000/µL at any point during their hospitalization and prior to having an eye exam) and Group (B) included infants with no thrombocytopenia (platelet count was never <150,000/µL during their hospitalization).

### Data collection

Data were extracted from electronic medical records, including demographic information, clinical characteristics (gestational age (GA), birth weight (BW), sex, small for gestational age (SGA) status), and clinical outcomes (respiratory distress syndrome (RDS) requiring surfactant, non-invasive (bubble continuous positive pressure ventilation (bCPAP) and positive pressure ventilation (NIPPV), and invasive ventilation (conventional ventilation and high frequency oscillation (HFO) days, bronchopulmonary dysplasia (BPD), congenital or acquired sepsis, necrotizing enterocolitis (NEC), intraventricular hemorrhage (IVH), or periventricular leukomalacia (PVL). Platelet count was obtained from routine complete blood count (CBC) tests performed as part of the infants’ routine clinical care. The diagnosis and classification of ROP were based on standard ophthalmologic examinations by certified pediatric ophthalmologists.

### Statistical analysis

Chi-square test was used to calculate and compare frequencies. Odds ratios (OR) were calculated to assess the association between thrombocytopenia and the development of any grade ROP or severe ROP (grades 3 and above). Adjusted odds ratios (aOR) for the relationship between thrombocytopenia and ROP or severe ROP were computed using logistic regression models while controlling for confounding factors including GA, BW, sex, SGA status, RDS with intubation and surfactant therapy, bCPAP, NIPPV, conventional ventilation, and HFO days, BPD, sepsis, NEC, IVH, PVL, and blood products transfusions. To examine the correlation between platelet counts as a continuous variable and the severity of ROP, Spearman’s correlation coefficient analysis was utilized. All statistical tests were two-sided, and a *p* value of less than 0.05 was considered statistically significant.

## Results

A total of 305 VLBW infants were included in the study. The population consisted of 51% females and 48% Caucasians. Approximately 25% of the infants were categorized as SGA. The mean gestational age was 28.2 weeks, and the mean birth weight was 1012 g. A majority (71%) of the births were via Cesarean delivery. The median Apgar score at 5 min was 7. Factors associated with thrombocytopenia include low GA or BW, SGA status, maternal hypertension, delivery room intubation, severe RDS necessitating the use of surfactant therapy, labile blood pressure requiring the use of hydrocortisone, the length of oxygen, non-invasive and invasive mechanical days but not bubble CPAP days, anemia of prematurity, the need for frequent packed red cells transfusions, sepsis, NEC, IVH and PVL, see Table 1.

**Table 1:** Demographic, perinatal, and clinical characteristics associated with thrombocytopenia.

	Platelets < 150 K *n* = 167	Platelets ≥ 150 K *n* = 138	95% CI, *p* value
Female sex	47	57	1.5 (0.9–2.3), 0.08
Race/Ethnicity: Caucasians	45	51	0.04
African Americans	33	23	
Others	22	26	
Gestational Age*	27 (3)	29 (3)	<0.01
Birth Weight*	862 (313)	1162 (244)	<0.01
Small for GA status	28	15	2.2 (1.2–3.9), <0.01
Cesarean delivery	75	72	1.1 (0.7–1.9), 0.69
Maternal hypertension	42	29	1.8 (1.1–2.9), 0.02
Maternal diabetes	13	12	1.2 (0.6–2.3), 0.72
Maternal Chorioamnionitis	10	12	0.8 (0.4–1.6), 0.57
Maternal Steroids	92	93	0.8 (0.3–1.8), 0.66
Maternal MgSO4	86	83	1.3 (0.7–2.4), 0.52
Placental abruption	8	14	0.5 (0.2–1.0), 0.07
Apgar1*	4 (2)	5 (2)	<0.01
Apgar5*	6 (2)	7 (1)	<0.01
Delivery room intubation	56	19	5.4 (3.2–9.1), <0.01
Respiratory Distress Syndrome	99	96	3.1 (0.6–16), 0.25
Surfactant therapy	74	38	4.8 (2.9–7.8), <0.01
Hydrocortisone therapy	30	3	14 (5–41), <0.01
Oxygen days*	19	3	<0.01
Bubble CPAP days*	20 (19)	17 (19)	0.14
Non-invasive positive pressure days*	9 (17)	3 (9)	<0.01
Invasive Ventilation days*	17 (31)	3 (10)	<0.01
Apnea of prematurity	97	91	3.1 (1.1–8.9), 0.04
Caffeine therapy	94	84	2.9(1.4–6.5), <0.01
Pneumothorax	11	3	4.0 (1.3–12), <0.01
Anemia of prematurity	96	92	1.9 (0.7–5.3), 0.22
Packed red cells transfusions	67	22	7.3 (4.3–12), <0.01
Early or late sepsis	37	13	3.9 (2.2–7.0), <0.01
Necrotizing enterocolitis	13	3	4.8 (1.5–14), <0.01
Patent ductus arteriosus	45	18	3.6 (2.1–6.2), <0.01
Intraventricular hemorrhage	48	28	2.4 (1.5–3.9), <0.01
Periventricular leukomalacia	16	7	2.7 (1.2–6.0), 0.01
Chronic lung disease	36	9	5.8 (2.9–11), <0.01
Length of hospital stay	89 (68)	59 (38)	<0.01
Discharge weight*	2825 (1388)	2527 (814)	0.02
Discharge length*	47 (5)	46 (4)	0.08
Discharge head circumference*	33 (4)	32 (2)	0.01

All values are presented as percentages except * in mean (±standard deviation).

As shown in Table 2, infants with thrombocytopenia (Group A) exhibited a higher incidence of ROP of any grade compared to those without thrombocytopenia (Group B) (47% vs. 16%, *p* < 0.01). The adjusted odds ratio (aOR) for the development of ROP of any grade in infants with thrombocytopenia was 2.9 (95% CI: 1.2–7.5, *p* < 0.01). Severe ROP occurred in 20% of infants in Group A, while none of the infants in group B developed severe ROP (*p* < 0.01). Logistic regression analysis controlling for confounding factors confirmed the association between thrombocytopenia and ROP. The adjusted odds ratios remained statistically significant for the association of thrombocytopenia with both any grade of ROP and severe ROP.

**Table 2:** Association of retinopathy of prematurity with thrombocytopenia.

	Platelets < 150 K *n* = 167	Platelets ≥ 150 K *n* = 138	95% CI/ *p* value
Retinopathy of prematurity	47	16	2.9 (1.2–7.5), <0.01
Severe retinopathy of prematurity	20	0	n/a

**Fig. 1:**
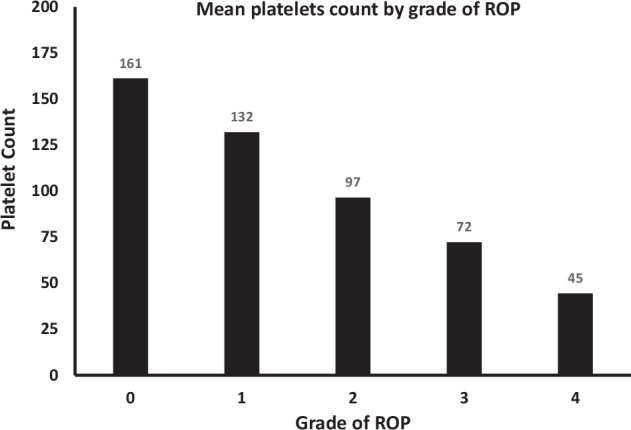
Mean lowest platelets count in infants who developed variable grades of retinopathy of prematurity.

The longitudinal correlation analysis revealed a significant inverse relationship between platelet count as a continuous variable and the grade of ROP, with a Spearman correlation coefficient indicating that the lower the platelet count the higher the grades of ROP: (CC: −0.36, *p* < 0.001), see relationship of platelets count and ROP in Fig. 1.

## Discussion

This study explored the association between thrombocytopenia and the development of ROP in VLBW infants, identifying a significant link that requires a deeper understanding of the underlying pathophysiology. The findings of our research highlight the role of thrombocytopenia as an independent factor in this association. Thrombocytopenia, generally defined as a platelet count of less than 150,000/µL, is a common occurrence in VLBW infants, and its implications for ROP have been increasingly recognized. In this study, thrombocytopenia was independently associated with the occurrence of ROP, mirroring the findings from Choręziak et al., who identified thrombocytopenia as a risk factor for the development and progression of ROP [3]. This suggests that the pathophysiological mechanisms underpinning thrombocytopenia may directly contribute to or exacerbate the vascular dysregulation characteristic of ROP.

Interestingly, our results also indicated an inverse correlation between the grade of ROP and the platelet count, suggesting that lower platelet counts are associated with higher grades of ROP. This inverse relationship aligns with the observation by Vinekar et al., who believed that platelets might play a role in the pathogenesis of aggressive posterior ROP through their involvement in angiogenic processes [7]. The involvement of platelets in angiogenesis, through the regulation of the VEGF, presents a plausible mechanism through which thrombocytopenia could influence ROP development, as hypothesized by Italiano et al. [8]

The involvement of platelets with transporting and activation of VEGF may explain its association with the development of ROP. Although not clearly understood or adequately studied, the relationship between transfusions and the potential unregulated release of cytokines is speculated to play a role in this process [9]. It is imperative to note that, the delivery of VEGF is physiologically regulated, and the fluctuation of the platelets level may be associated with fluctuation of the delivery or over exposure of VEGF. Whether this physiologic regulation of VEGF expression applies for transfused platelets is unclear. In, a meta-analysis including seventeen studies utilized ELISA to examine VEGF as a biomarker for ROP, revealed antagonizing outcomes. While eight studies associated higher serum VEGF levels with ROP, nine found no significant differences in VEGF concentrations between infants with and without ROP. Another meta-analysis of 11 studies showed a standardized mean difference (SMD) of −0.07 [−1.06, 0.93], indicating potentially lower VEGF levels in infants with ROP, yet the results were highly variable and inconclusive due to the confidence interval crossing the neutral line. Additionally, studies on VEGFR-1 and VEGFR-2, evaluated in six studies via ELISA, found no significant differences in levels related to ROP development. [8]

Moreover, our study highlights the significance of timely and rigorous screening for thrombocytopenia in VLBW infants, given its potential association with ROP. This is particularly crucial given the findings of Lundgren et al., which emphasized the correlation between multiple infectious episodes, thrombocytopenia, and the aggressive form of ROP [10]. These results show the multifactorial nature of ROP, where thrombocytopenia interplays with other risk factors, such as infections, to influence the disease’s onset and progression.

Further, the association of red cells transfusion with ROP continues to be an area of ongoing research. Although, the exact mechanism is still unclear, there has growing evidence suggest that red blood cell transfusion increases the oxygen load on the developing retina and might contribute to the development of ROP [9, 11–14]. Mechanistic studies suggest the reason for this association involves adult hemoglobin releasing higher amounts of oxygen to the developing retina; contrarywise, fetal hemoglobin releases less and therefore is protective against ROP [13–16]. Infants with thrombocytopenia often require transfusions due to the concurrent anemia intensifying oxidative stress and oxygen fluctuations, both of which contribute to ROP pathogenesis. Controlling for this confounder, our study demonstrates that thrombocytopenia remains an independent risk factor for ROP in preterm infants. However, caution must be exercised in infants while transfusing a preterm infant with thrombocytopenia. Further studies are required to explore the interplay and the causal mechanisms between red blood transfusion, thrombocytopenia and ROP development.

The study’s findings contribute to the growing body of evidence that thrombocytopenia might not only be a marker of illness severity but also a potential modifiable risk factor in the pathogenesis of ROP. This association opens new avenues for research into the mechanisms by which platelets and thrombocytopenia may influence retinal vascular development and the progression of ROP. Understanding these pathways could lead to new prevention and treatment strategies, potentially improving outcomes for this vulnerable population.

### Limitations

This study’s findings of the association of thrombocytopenia with ROP in VLBW infants must be considered within the context of its limitations. Being retrospective in nature, our analysis might be influenced by biases inherent to retrospective data collection and documentation. Because the study was conducted within a single hospital network, the generalizability of our results to other settings may be limited. Clinical practices, demographic characteristics, and healthcare resources can vary significantly across different regions and institutions. While adjustments were made for several known confounders, the possibility of residual confounding be un- or inadequately measured remains high. Particularly, factors like nutritional status, specific neonatal treatments or intervention, and the timing of platelet transfusions that were not fully accounted for.

The definition of thrombocytopenia as a platelet count below 150,000/µL, though common in clinical practice, does not account for the potential differential impact of varying degrees of thrombocytopenia on ROP risk. It also does not distinguish between transient versus persistent thrombocytopenia. Moreover, the study’s design did not allow for the capture of longitudinal platelet data throughout the neonatal period. This possibly overlooks the fluctuations in platelet counts and their temporal relationship with the progression of ROP.

Finally, infants with thrombocytopenia or other morbidities might undergo more frequent ophthalmologic evaluations, introducing surveillance bias in the diagnosis of ROP.

These limitations highlight the need for caution in interpreting the study’s results and the need for future prospective studies to validate these findings and investigate underlying mechanisms.

## Conclusion

This is the first study to correlate the degree of thrombocytopenia with the severity of ROP. The findings from this study show a significant association between thrombocytopenia and the development of ROP in VLBW infants. Our results highlight that thrombocytopenia is associated with an increased risk of both any grade ROP and severe ROP, independent of other known risk factors such as GA, BW, sex, and the presence of other neonatal morbidities like RDS, BPD, sepsis, NEC, IVH, or PVL. Furthermore, the significant inverse correlation observed between platelet counts and the severity of ROP underscores the importance of closely monitoring platelet levels in VLBW infants and investigating the benefits of interventions aimed at stabilizing platelet counts. However, it is crucial to approach such interventions with caution, as the therapeutic implications of these findings require validation in prospective, controlled clinical trials to ascertain the effectiveness and safety of manipulating platelet counts in the prevention or management of ROP. Future studies should aim to investigate the underlying mechanisms of this association and explore targeted interventions that may mitigate the risk of ROP development in this high-risk population.

## Data Availability

The data supports the findings of this study are available at the Division of Neonatology, Department of Pediatrics, Children’s Institute, Cleveland Clinic Foundations. It can be requested by emailing the corresponding author at mohamem2@ccf.org.
